# Preparation of a Novel Lignocellulose-Based Aerogel by Partially Dissolving Medulla Tetrapanacis via Ionic Liquid

**DOI:** 10.3390/gels10020138

**Published:** 2024-02-09

**Authors:** Long Quan, Xueqian Shi, Jie Zhang, Zhuju Shu, Liang Zhou

**Affiliations:** 1 School of Materials and Chemistry, Anhui Agricultural University, Hefei 230036, China; longquan_ahau@163.com (L.Q.); 15357108782@163.com (X.S.); zhangjie@goldenchemical.com (J.Z.); 2 Key Lab of State Forest and Grassland Administration on Wood Quality Improvement & Utilization, Hefei 230036, China

**Keywords:** Medulla tetrapanacis, lignocellulose-based aerogel, ionic liquid, absorption of dye

## Abstract

A novel lignocellulosic aerogel, MT-LCA, was successfully prepared from MT by undergoing partial dissolution in an ionic liquid, coagulation in water, freezing in liquid nitrogen, and subsequent freeze-drying. The MT-LCA preserves its original honeycomb-like porous structure, and the newly formed micropores contribute to increased porosity and specific surface area. FT-IR analysis reveals that MT, after dissolution and coagulation, experiences no chemical reactions. However, a change in the crystalline structure of cellulose is observed, transitioning from cellulose I to cellulose II. Both MT and MT-LCA demonstrate a quasi-second-order kinetic process during methylene blue adsorption, indicative of chemical adsorption. The Langmuir model proves to be more appropriate for characterizing the methylene blue adsorption process. Both adsorbents exhibit monolayer adsorption, and their effective adsorption sites are uniformly distributed. The higher porosity, nanoscale micropores, and larger pore size in MT-LCA enhance its capillary force, providing efficient directional transport performance. Consequently, the prepared MT-LCA displays exceptional compressive performance and efficient directional transport capabilities, making it well-suited for applications requiring high compressive performance and selective directional transport.

## 1. Introduction

Abundant storage stems of plants supply enormous quantities of lignocellulosic resources [1]. To deal with the problems arising from fossil-based energy and products, the lignocellulosic materials are attracting greater attention due to their biodegradable, low-cost, and versatile applications [1,2]. Since the living stems of plants play a vital role in transporting water from the earth to the crown or leaves and mechanical support, the structure of the stem is assembled in a porous and hierarchical manner built mainly of cellulose, hemi-cellulose, and lignin [3]. Therefore, various kinds of stem directly or indirectly, namely after certain adjustments or modifications, are used as porous functional materials [4]. *Tetrapanax papyriferus*, is a typical fast-growing bush with large leaves and its natural range is in southern China [5]. The stem pith of the plant, which is known as Medulla tetrapanacis (MT), is a traditional Chinese medicine called “da-tong-cao”. It is considered helpful in promoting diuresis and alleviating edema and abdominal distention [6]. Moreover, MT also is a traditional resource for preparing painting paper, since it has a white color and reasonable luster. Recently, MT has been pyrolyzed into porous carbon materials for multiple applications as an absorbent or supercapacitor electrode [7]. It turns out that the original porous structure in itself is beneficial for the required properties of the output products [8].

Lignocellulose-based aerogels have been a subject of increasing interest in materials science and sustainable technologies, since they can replace or partially replace aerogels originated from synthetic polymers [9]. These aerogels are lightweight, highly porous materials made from lignocellulosic biomass sources such as wood, agricultural residues, and paper waste. They have a wide range of potential applications due to their biodegradability, low cost, and eco-friendliness [10,11]. Lignocellulose-based aerogels have demonstrated potential in the removal of dyes and pigments from aqueous solutions, making them useful in environmental remediation, wastewater treatment, and water purification [12,13]. Compared to the common synthetic polymer sorbents, the lignocellulosic aerogel is more environmentally friendly, biodegradable, and relatively low-cost [14,15]. In addition, heat or sound insulation, storage of energy, catalysis support and other versatile applications of lignocellulosic aerogels have also been reported recently. Lignocellulosic aerogels are generally prepared from wood, sourced through two independent strategies. The first one is dehydration from hydrogel, which is mostly harvested by coagulating the dissolving cellulose or lignocellulose solution, by freeze-drying or supercritical drying. The second strategy is to construct them with nanofilaments, which generally are composed of cellulose and lignocellulose. Moreover, Garemark proposed a different method to produce hierarchical and anisotropic lignocellulosic aerogel by keeping the main microstructural porous of wooden stem intact and constructing aerogels inside the lumen simultaneously by partially dissolving and coagulating cell walls [16]. It is found that such a structure is beneficial to fabricating anisotropic cellulose aerogels and can be considered as a universal substrate in multiple fields.

As mentioned earlier, Medulla tetrapanacis (MT) exhibits a characteristic porous microstructure, suggesting that its density should be lower than that of the wooden stem, as observed in Garemark’s development of a lignocellulosic aerogel [16]. This observation served as an inspiration for us to adopt a similar approach in crafting anisotropic lignocellulosic aerogel from MT. To partially dissolve the MT efficiently, one kind of EmimOAc was selected, since it was reported that it had good dissolving ability not only for cellulose but also for lignocellulosic resources and it can maintain relatively low viscosity at room temperature, which benefits permeation [14]. Subsequently, we subjected the aerogel to testing to evaluate its capacity for adsorbing dyes or pigments from water. Additionally, we investigated the adsorption kinetics of the aerogel to comprehend the adsorption process. To elucidate the mechanism of adsorption, we conducted examinations of the chemical structure, microstructure, porosity, and thermal stability. We anticipate that the anisotropic lignocellulosic aerogel derived from MT through this method holds potential applications in both dye removal from water solutions and pigment adsorption in organic solvents. This development not only enhances the value of MT but also provides an economical alternative for anisotropic lignocellulosic aerogel in the field of environmental protection.

## 2. Results and Discussion

### 2.1. Morphological Features

**Figure 1:**
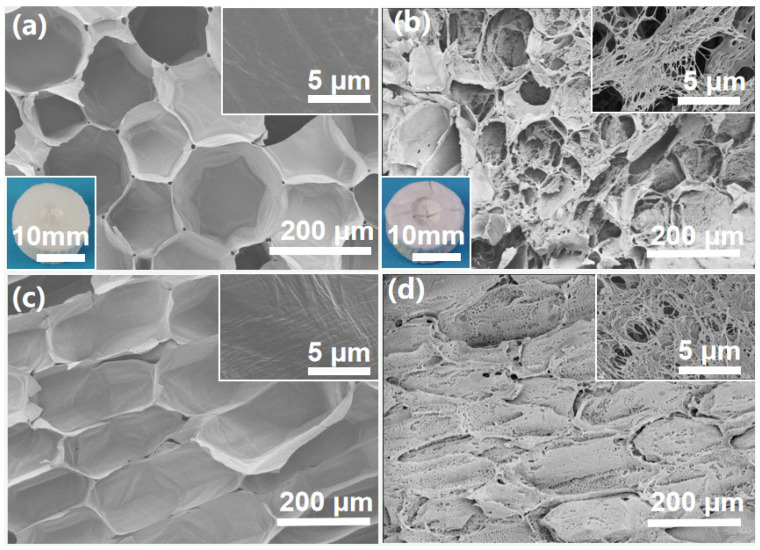
SEM pictures of MT and MT-LCA: (**a**) Cross section of MT, (**b**) Cross section of MT-LCA, (**c**) Tangential section of MT, (**d**) Tangential section of MT-LCA.

To investigate the morphological changes of MT and MT-LCA before and after EmimOAc treatment, SEM observations were conducted. Figure 1a shows the cross-sectional SEM image of MT, which inherently possesses a honeycomb-like porous structure with an average pore size of approximately 150 μm and an average pore wall thickness of around 1 μm. Figure 1c depicts the longitudinal section of MT, revealing a well-organized orientation of pore channels. Compared to the transverse direction, the longitudinal pores in MT are larger, presenting a more open and uniform porous structure. The abundant porous structure of MT contributes to its high porosity (40%) and low density (0.05 g/cm^3^). This characteristic not only facilitates rapid infiltration of EmimOAc into the pores during dissolution but also shortens the dissolution time while ensuring a more uniform dissolution process.

Figure 1b and Figure 1d, respectively, depict the cross-section and tangential-section SEM images of MT-LCA. MT-LCA not only retains the original transverse honeycomb-like porous structure and longitudinally oriented pore channels of MT but also exhibits an increased number of pores in the original cell walls. Simultaneously, a fibrous network structure appears within the cell lumens, creating numerous tiny pores. Under the effects of EmimOAc dissolution and swelling, the original cell wall structure of MT undergoes disruption, leading to the dissolution of some natural polymers in the EmimOAc solution. This dissolution results in the etching of the cell walls, forming distinct new pores. The natural polymers dissolved in the EmimOAc solution enter the cell lumens and, after coagulation with deionized water, form a hydrogel. Rapid freezing with liquid nitrogen forms small-sized ice crystals within the hydrogel. Following freeze-drying, both the pores created by EmimOAc etching and those formed by ice crystals are preserved.

Therefore, MT-LCA has been successfully prepared from MT through being partially dissolved in an ionic liquid, coagulated in water, frozen in liquid nitrogen and freeze-dried. It retains the original transverse honeycomb-like porous structure and longitudinally oriented pore channels while acquiring smaller micropores. The preservation of anisotropic structures imparts excellent compressive performance and efficient adsorption rates to MT-LCA. The newly generated micropores will contribute to an increased porosity in MT-LCA, enhancing its capability to adsorb dye molecules and other solvents [17].

### 2.2. FTIR Analysis

**Figure 2:**
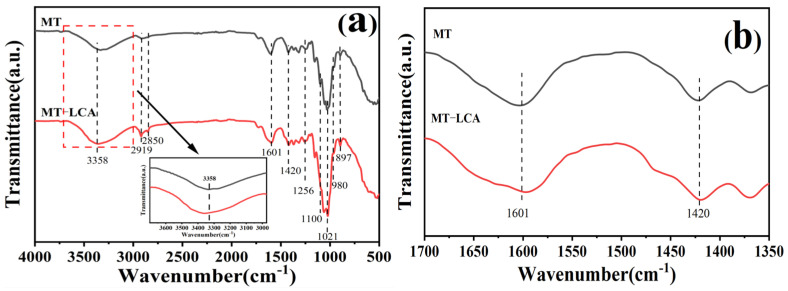
(**a**) FTIR spectra of MT and MT-LCA, (**b**) Magnified image from a to show the area between 1350 to 1700 cm^−1^.

The infrared spectra of MT and MT-LCA are shown in Figure 2. The infrared spectra of MT and MT-LCA are quite similar, with no significant changes observed in the positions of the main absorption peaks. This suggests that during the process of dissolution and regeneration, there were no chemical reactions occurring in the lignocellulosic fibers of MT, and the molecular vibrational modes remained unchanged.

As shown in Figure 2a, the absorption peak around 3358 cm^−1^ corresponds to the -OH stretching vibration of cellulose in both MT and MT-LCA. In MT-LCA, the absorption peak at 3358 cm^−1^ shifts to a higher wavenumber, indicating that the hydrogen bonding within and between the original natural polymers (cellulose, hemicellulose, and lignin) in Medulla tetrapanacis (MT) has been somewhat disrupted, leading to a weakening of the hydrogen-bonding interactions after partial dissolution in EmimOAc [18]. The peaks at 2919 cm^−1^ and 2850 cm^−1^ correspond to the C-H stretching vibrations of cellulose. The absorption peak at 1601 cm^−1^ corresponds to the aromatic ring skeletal vibration of lignin, while the peak at 1100 cm^−1^ is associated with the syringyl propane structural unit in lignin [19]. Both peaks in MT-LCA are weakened, indicating a lower lignin content in MT-LCA compared to MT, possibly due to partial degradation of lignin during the dissolution and regeneration process. The infrared absorption peak at 1420 cm^−1^ is related to the crystalline spectrum of cellulose I (Figure 2b). The reduction in the characteristic absorption peak at 1420 cm^−1^ suggests a decrease in the cellulose I content in MT-LCA, possibly due to the dissolution of natural cellulose in MT [20]. The peaks at 1256 cm^−1^ and 897 cm^−1^ correspond to the C-O stretching vibration of cellulose and the characteristic absorption peak of the β-O-4 glycosidic bond in hemicellulose, respectively. These peaks show no significant changes after dissolution and regeneration. Through infrared spectroscopy, a comparison of the chemical structures of Medulla tetrapanacis (MT) before and after dissolution reveals a weakening of hydrogen bonds in the original natural polymers, partial degradation of lignin, and a transition of cellulose from a crystalline I structure to II structure in MT-LCA [21].

### 2.3. XRD Analysis

**Figure 3:**
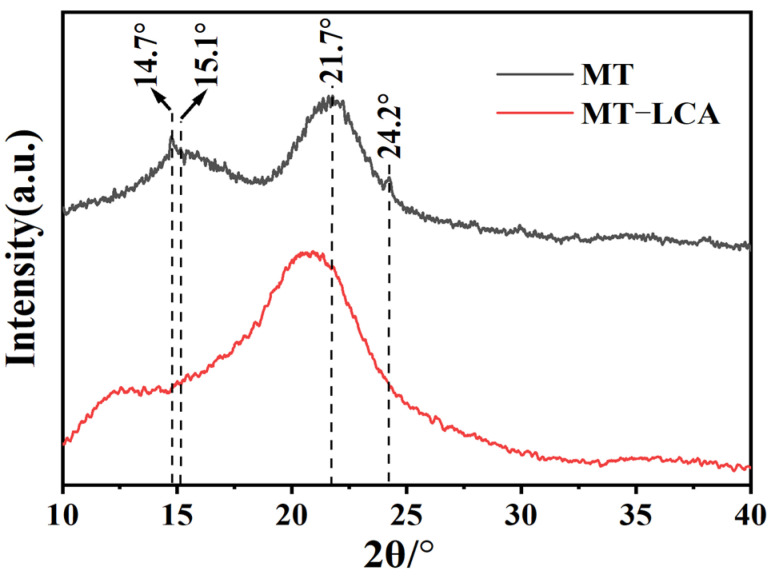
XRD patterns of MT and MT-LCA.

To investigate the crystalline structure of MT and MT-LCA, X-ray diffraction (XRD) was employed for sample characterization. The XRD test results for MT and MT-LCA are shown in Figure 3, where MT exhibits four diffraction peaks distributed at 14.7°, 15.1°, 21.7°, and 24.2°.

The diffraction peaks at 15.1° and 21.7° correspond to characteristic cellulose I-type diffraction peaks, representing the 101 and 200 crystallographic planes of cellulose [22], indicating that MT possesses a typical cellulose I crystalline structure. The diffraction peaks at 14.7° and 24.2° are characteristic diffraction peaks of calcium salts, suggesting the presence of a small amount of calcium salts in MT. In comparison to MT, the positions of the diffraction peaks in MT-LCA, prepared through dissolution and regeneration, have changed [4]. As shown in Figure 3, the diffraction peak at 21.7° in MT shifts to 20.6° after dissolution and regeneration. This shift indicates a transformation of the cellulose crystalline structure from the original cellulose I to cellulose II type [23]. For the same reason, the diffraction peak at 15.1° in MT also shifts to 12.5°. Comparing the XRD curves of MT and MT-LCA, the characteristic peaks related to calcium salts at 14.7° and 24.2° disappear after dissolution and regeneration, suggesting that calcium salts are dissolved and removed from the material during dissolution and washing processes [4].

### 2.4. Thermal Stability

**Figure 4:**
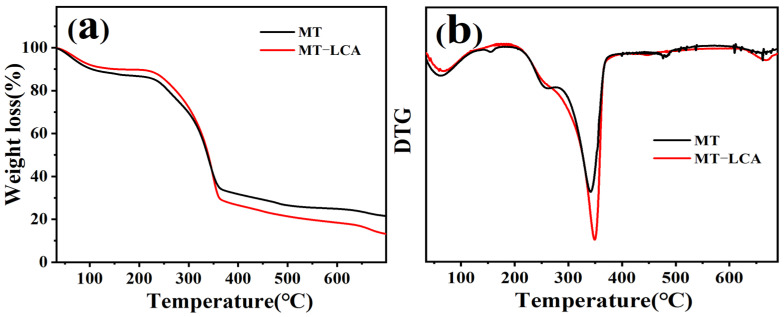
Thermogravimetric curves of MT and MT-LCA: (**a**) TGA curves, (**b**) DTG curves.

To compare the thermal stability of MT and MT-LCA, thermogravimetric (TG) testing was conducted [24], and the results along with a differentiated thermogravimetric (DTG) curve are shown in Figure 4 and Table 1. The thermal weight loss curves of MT and MT-LCA are generally similar, indicating that their thermal decomposition patterns are roughly the same. The first stage, ranging from 30 °C to 100 °C, primarily involves the desorption weight loss of physically adsorbed water in wood cellulose and the removal of water molecules bound by hydrogen bonding in cellulose [25]. The second stage, occurring from 230 °C to 400 °C, involves the sequential thermal decomposition of hemicellulose and cellulose. As the temperature gradually rises, the rate of thermal weight loss accelerates, with the β-O-4 ether bonds of some glucose units in the cellulose main chain breaking [26], followed by the cleavage of the pyranose rings. The third stage, occurring after 400 °C, involves the decarboxylation of cellulose, leading to the formation of carbon black and the release of volatile gases [27]. During this stage, lignin also begins to undergo thermal degradation [28].

Upon careful comparison of the thermogravimetric curves of MT and MT-LCA, it is observed that the initial thermal decomposition temperature of cellulose in MT-LCA is lower, and its decomposition rate is faster compared to MT. This suggests that the thermal stability of MT-LCA is lower. The reason for this phenomenon may be attributed to the disruption of the crystalline structure of cellulose in MT-LCA after dissolution and regeneration [29]. As the temperature increases, the amorphous region of cellulose in MT-LCA begins to decompose. In comparison to the natural cellulose in MT, the regenerated cellulose in MT-LCA exhibits lower crystallinity, with a larger proportion of the amorphous region. The thermal stability of cellulose decreases with a reduction in crystallinity [30]. Through a comparison of the thermogravimetric curves, it is evident that MT-LCA has lower thermal stability relative to MT.

### 2.5. Compressive Tests

**Figure 5:**
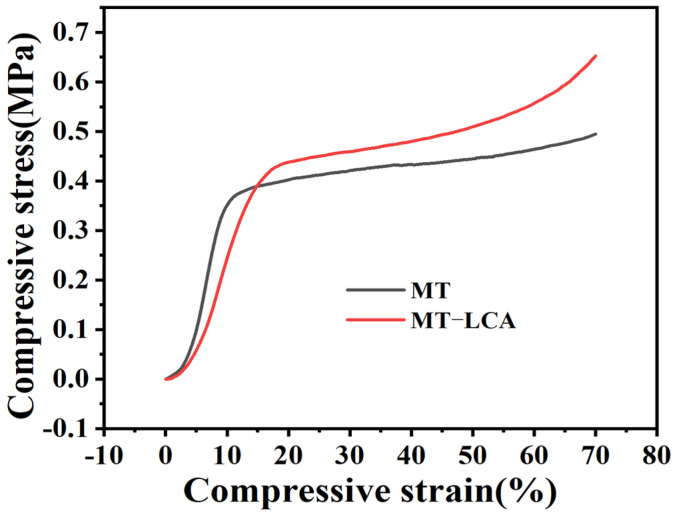
Compression stress–strain curves of MT and MT-LCA.

To investigate the compression performance of MT and MT-LCA, mechanical compression experiments were conducted. Figure 5 depicts the compression stress–strain curves for MT and MT-LCA.

MT and MT-LCA undergo three stages during compression: the first stage involves a linear increase in compression stress with increasing compression strain in the elastic phase when the compression strain is relatively small. When the strain exceeds the yield strength, the regular pore structure within MT and MT-LCA starts to collapse. The internal network fibers come into contact and squeeze against each other, leading to plastic deformation in MT and MT-LCA. During this stage, as strain increases, stress increases more gradually and approaches a plateau, representing the yielding platform in the second stage. The third stage is the densification stage, where with continuous compression deformation of MT and MT-LCA, stress sharply increases, and the regular honeycomb-like pore structure is severely disrupted, indicating a trend toward densification of the internal three-dimensional network structure [31].

Compared to MT, MT-LCA has some of the natural polymers in the cell wall dissolved by EmimOAc, causing a certain degree of damage to its pore structure, resulting in reduced compression strength in the initial stage. As shown in Table 2, MT-LCA has a higher density than MT (densities of MT-LCA and MT are 0.084 g/cm^3^ and 0.050 g/cm^3^, respectively). With increasing compression strain, the stronger intermolecular forces in the higher-density MT-LCA contribute to its superior compressive performance compared to MT. Although MT-LCA exhibits slightly lower mechanical strength than MT in the initial stage, it outperforms cellulose aerogels prepared from bottom to top. MT-LCA retains the original honeycomb-like pore structure and regularly oriented pore channel structure of MT, while other cellulose aerogels, lacking honeycomb-like pore structures and regularly oriented pore channels, easily experience pore structure collapse and show inferior compression strength under pressure [19].

### 2.6. Porosity Analysis

**Figure 6:**
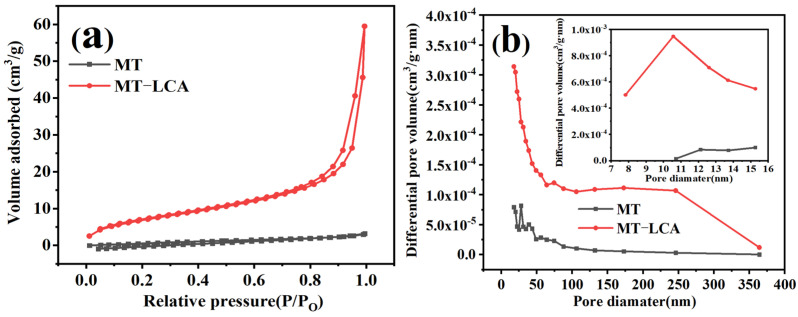
Nitrogen adsorption–desorption curves and pore size distribution diagram of MT and MT-LCA: (**a**) Nitrogen adsorption–desorption curves, (**b**) Pore distribution diagram.

To investigate the changes in specific surface area before and after the partial dissolution of MT, nitrogen adsorption–desorption tests were conducted on MT and MT-LCA. The nitrogen adsorption–desorption curves for MT and MT-LCA are shown in Figure 6. The IUPAC classifies physical adsorption isotherms into six types, and the nitrogen adsorption–desorption curves of MT and MT-LCA fall into Type II, characteristic of mesoporous adsorbent materials with interactions between adsorbent and adsorbate. The presence of an H3 hysteresis loop indicates the existence of narrow mesoporous structures (2–50 nm) [4,5,7].

At low pressures, the adsorption behavior of MT-LCA is mainly monolayer adsorption, transitioning to multilayer adsorption as the pressure increases. Comparing the nitrogen adsorption–desorption curves of MT and MT-LCA, at low relative pressures (<0.01), MT-LCA shows a distinct upward trend, indicating the presence of numerous micropores. In contrast, the curve for MT remains relatively unchanged, suggesting the absence of micropores in untreated MT. When the relative pressure exceeds 0.8 and the adsorbed N_2_ transforms into a liquid state, the nitrogen adsorption–desorption curve for MT-LCA sharply rises, indicating the presence of some large pores where nitrogen condenses, causing the curve to increase [32].

The nitrogen adsorption–desorption curves were used to calculate their porosity, total pore volume, average pore diameter, and specific surface area, with the results shown in Table 3. The porosity of MT-LCA increases to 51%, and the average pore diameters of MT and MT-LCA are 36.71 nm and 22.17 nm, respectively. The total pore volume and specific surface area of MT are only 0.001 cm^3^/g and 0.669 m^2^/g, significantly lower than those of MT-LCA.

To further compare the pore structures of MT and MT-LCA, pore size distribution calculations were performed using the BJH and HK models [4,8]. The pore size distribution results are shown in Figure 6b. In MT, the mesopore diameters are primarily concentrated in the range of 10 to 16 nm, and the macropore diameters are mainly in the range of 50 to 100 nm. However, the quantities of micropores, mesopores, and macropores in MT are relatively low. In contrast, MT-LCA exhibits a higher abundance of micropores, mesopores, and macropores. Additionally, the distribution ranges of mesopore and macropore diameters in MT-LCA are broader compared to MT. The mesopore diameters in MT-LCA are mainly distributed in the range of 8 to 16 nm, and the number of macropores with diameters in the range of 50 to 250 nm is also higher than in MT.

The changes in the pore structures described above are consistent with the observations from SEM. After dissolution and swelling with EmimOAc, the original cell wall structure of MT is disrupted. Some cellulose and hemicellulose dissolve in the EmimOAc solution, resulting in etching of the cell wall and the formation of new pores. The cellulose and hemicellulose dissolved in the EmimOAc solution enter the cell cavity and, after deionized water coagulation, form nano-fibers that aggregate into a hydrogel [15]. Rapid freezing with liquid nitrogen at this stage forms smaller ice crystals inside the hydrogel. After freeze-drying, the pores formed by the EmimOAc solution etching and the pores formed by the ice crystals in the hydrogel are retained. This is likely the main factor contributing to the improvement of the pore structure-related data for MT-LCA.

### 2.7. Absorption of Dye

**Figure 7:**
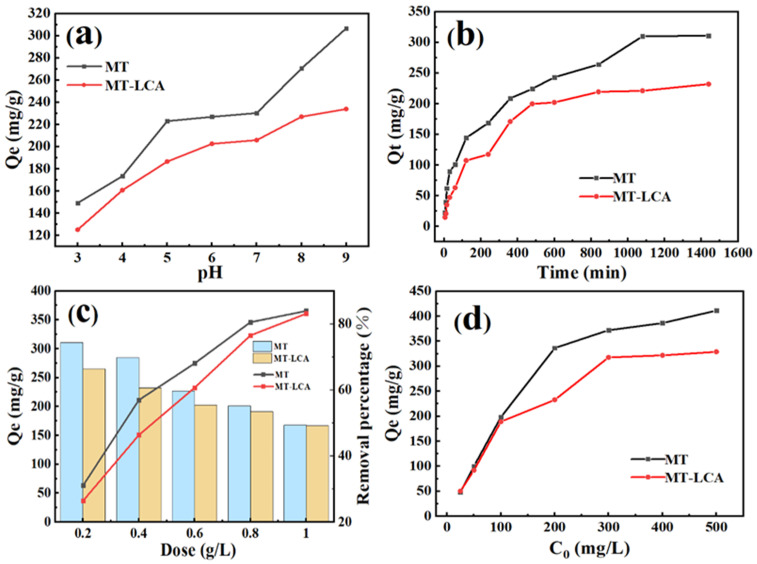
The effect of different conditions on the adsorption performance of adsorbents: (**a**) Solution pH value, (**b**) Adsorption time, (**c**) Amount of adsorbent added, (**d**) Initial concentration of solution.

Methylene blue, as a model dye, was used to compare the adsorption capacity of MT and MT-LCA for dyes, and the results are shown in Figure 7. To investigate the influence of solution pH on the adsorption performance of the adsorbents, the adsorption amounts of MT and MT-LCA for methylene blue were tested at different pH values. The relationship curve between solution pH values and adsorption amounts is depicted in Figure 6a. As the pH values gradually increase, the adsorption trend of both MT and MT-LCA for methylene blue solution is generally similar, showing a gradual increase. This is mainly because MT and MT-LCA contain carboxyl and hydroxyl groups, and the acidity and alkalinity of the solution affect the charge of these two functional groups in the aqueous solution [33]. When the solution is acidic, the adsorbent carries a positive charge, while methylene blue exists in the solution in cationic form. With a large number of H^+^ ions in the solution occupying the surface of MT and MT-LCA, electrostatic repulsion occurs with the cationic form of methylene blue in the solution, resulting in a lower adsorption amount at lower pH values. However, as the pH value rises, and the solution becomes alkaline, the adsorbent’s charge transforms into a negative charge. This leads to electrostatic attraction between the adsorbent and methylene blue dye, gradually increasing the adsorption amount [34].

To investigate the effect of adsorption time on the adsorption performance of MT and MT-LCA for methylene blue, the adsorption amounts of MT and MT-LCA for methylene blue were tested at different time intervals. The relationship curve between adsorption time and adsorption amount is shown in Figure 7b. Before reaching adsorption equilibrium, the adsorption amounts of methylene blue by both MT and MT-LCA increase with the extension of adsorption time. When the adsorption time is in the range of 0 to 400 min, the adsorption amount of the adsorbent increases rapidly. However, beyond 400 min, the growth of the adsorption amount becomes more gradual. Eventually, MT reaches adsorption equilibrium around 1100 min, while MT-LCA achieves adsorption equilibrium around 850 min. This is because, in the initial stage of adsorption, the surfaces of MT and MT-LCA have numerous adsorption sites, and there is a significant difference in methylene blue concentration between their interiors and exteriors, facilitating the rapid diffusion and attachment of methylene blue molecules to the adsorbent. As time progresses, the adsorption sites on the surface of the adsorbent are gradually occupied by methylene blue molecules, leading to a gradual reduction in the adsorption rate [35]. Ultimately, the effective adsorption sites of the adsorbent are fully occupied, and the adsorption capacity of MT and MT-LCA for dye molecules reaches saturation, achieving dynamic equilibrium in the adsorption and desorption of dye molecules by MT and MT-LCA [32].

To investigate the impact of adsorbent dosage on the adsorption performance of MT and MT-LCA for methylene blue, tests were conducted with different dosages of MT and MT-LCA to measure the adsorption amount and removal efficiency of methylene blue. The relationship between adsorbent dosage, removal efficiency, and adsorption amount is shown in Figure 7c. As the dosage of MT and MT-LCA increases, more adsorbent provides additional adsorption sites, facilitating the adsorption of more methylene blue molecules. Therefore, the removal efficiency of methylene blue by the adsorbent increases with the rising dosage of MT and MT-LCA. However, as the concentration of methylene blue in the solution decreases and adsorption approaches saturation, the adsorption sites of the adsorbent cannot be fully utilized [35]. Consequently, when the dosage increases to a certain extent, the increase in removal efficiency becomes more gradual. The adsorption amount and removal efficiency exhibit an inverse trend. With an increasing dosage of the adsorbent, the adsorption amount of MT and MT-LCA for methylene blue continuously decreases. This is due to an excessive number of adsorption sites in the solution, leading to increased competition among more adsorbents for the adsorption of dye molecules and resulting in a reduction in the adsorption amount per unit mass of adsorbent for dye molecules [34].

To investigate the impact of initial solution concentration on the adsorption performance of MT and MT-LCA for methylene blue, tests were conducted with MT and MT-LCA at different initial concentrations of methylene blue, and the relationship between the initial concentration of the solution and the adsorption amount is shown in Figure 7d. With an increase in the initial concentration of methylene blue solution, the adsorption amount of both MT and MT-LCA also increases, but the trend gradually levels off. The main reason for this is that as the concentration of methylene blue in the solution increases, the concentration difference between the solution and the adsorbent becomes larger, driving more methylene blue molecules into the interior of the adsorbent, where they combine with the adsorption sites of MT and MT-LCA. However, the effective adsorption sites on the adsorbent are limited. Therefore, as the concentration of methylene blue solution continues to increase, the effective adsorption sites of the adsorbent are almost entirely occupied by methylene blue molecules, leading to a tendency of the adsorption amount by the adsorbent for methylene blue solution to approach equilibrium [34,35].

### 2.8. Absorption Mechanism of Dye

In order to investigate the adsorption mechanisms of MT and MT on the aqueous solution of methylene blue, adsorption dynamic models were employed to predict the relationship between adsorption amount and adsorption time. This approach was utilized to explore the adsorption mechanisms involved in the process of MT and MT adsorbing methylene blue.

**Figure 8:**
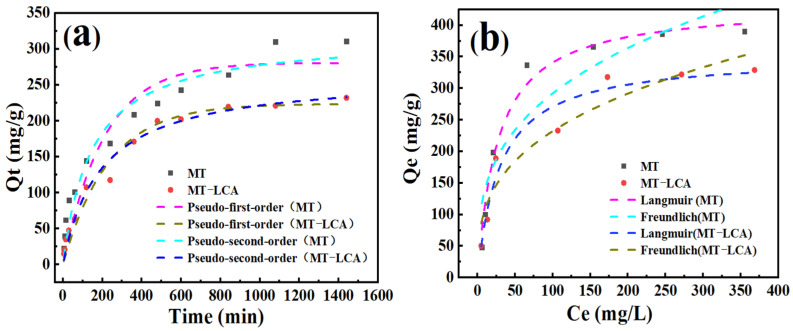
Kinetic adsorption fitting curves (**a**) and adsorption isotherms (**b**) of MT and MT-LCA.

The dynamic simulation results obtained from Equations (3) and (4), as well as the adsorption fitting curves of the dynamic models, are shown in Figure 8 and Table 4. The correlation coefficients obtained from the quasi-second-order kinetic model for both MT and MT-LCA are higher than those obtained from the quasi-first-order kinetic model. Particularly, the correlation coefficient for the quasi-second-order kinetic model of MT-LCA reaches up to 0.99, providing a more accurate description of the adsorption process of methylene blue molecules by MT-LCA. The adsorption equilibrium amount obtained from the quasi-second-order kinetic model for MT-LCA also closely matches the experimentally measured adsorption amount. Consequently, the adsorption process of both MT and MT-LCA on methylene blue is better described by the quasi-second-order kinetic model. This indicates that the adsorption of methylene blue by MT and MT-LCA is primarily a chemical adsorption process, and the adsorption capacity of MT and MT-LCA is positively correlated with their adsorption sites [36]. The adsorption process involves both surface adsorption and internal diffusion [37].

To investigate the interaction between MT, MT-LCA, and methylene blue, the Langmuir isotherm equation (Equation (5)) and Freundlich isotherm equation (Equation (6)) were employed to fit the data of methylene blue adsorption by MT and MT-LCA at different initial concentrations. The obtained adsorption isotherms and isotherm parameters are presented in Figure 8 and Table 4. In comparison to the correlation coefficients of the Freundlich model adsorption isotherms (0.84 and 0.90), the correlation coefficients of the Langmuir model adsorption isotherms for MT and MT-LCA (0.98 and 0.95) are higher. This suggests that the Langmuir model is more suitable for describing the adsorption of methylene blue by MT and MT-LCA [38]. Therefore, both adsorbents exhibit monolayer adsorption of methylene blue, and their effective adsorption sites are uniformly distributed on their surfaces.

### 2.9. Absorption of Oil

**Figure 9:**
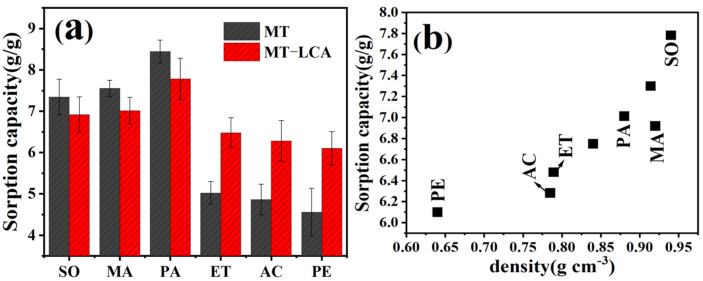
Saturated adsorption capacities of MT and MT-LCA: (**a**) Saturated adsorption capacities of MT and MT-LCA for different oils, (**b**) Relationship between saturated adsorption capacities of MT-LCA and oil density.

To further analyze the adsorption performance of MT and MT-LCA, their saturation adsorption capacities were tested for six different oils and organic solvents [30,39], as shown in Figure 9. For viscous oils such as soybean oil, engine oil, and paraffin oil, MT-LCA performs slightly worse than MT, but the difference is not significant. However, MT-LCA exhibits a significantly higher saturation adsorption capacity for organic solvents compared to MT. Due to its higher density, MT-LCA has a smaller volume than MT under the same conditions. Therefore, when immersed in viscous oils, the contact area of MT-LCA with the oil is smaller than that of MT. Meanwhile, since viscous oils provide high saturation adsorption capacity through surface adhesion, MT achieves a higher saturation adsorption capacity [40]. When MT and MT-LCA are immersed in organic solvents with lower viscosity, the volume advantage of MT is less pronounced. Additionally, organic solvents can migrate to the interior through surface tension, utilizing the material’s porous structure. Given that the porous structure of MT-LCA is superior to MT, its saturation adsorption capacity for organic solvents is significantly higher than that of MT.

To further explore the adsorption mechanism of MT-LCA for oils, we analyzed the relationship between the saturation adsorption capacity of the reed cellulose aerogel and the density of oils and organic solvents. As shown in Figure 9b, the saturation adsorption capacity of MT-LCA for oils and organic solvents is positively correlated with the density of oils and organic solvents. The higher the density of oils and organic solvents, the greater the saturation adsorption capacity of MT-LCA. This is primarily because, with a constant volume of the adsorbent, the number of effective adsorption sites is fixed, and the volume change range for adsorbing different types of oils and organic solvents is relatively small [30]. Therefore, the higher the density of oils and organic solvents, the greater the mass of oils and organic solvents that the adsorbent can adsorb [30,39].

**Figure 10:**
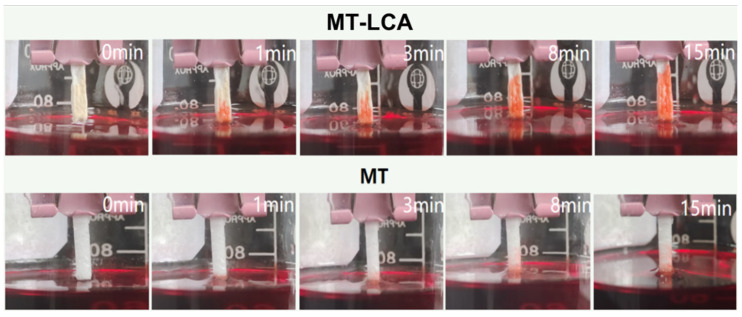
Absorbing behavior of the MT and MT-LCA in soybean oil (Sudan III dyed).

**Figure 11:**
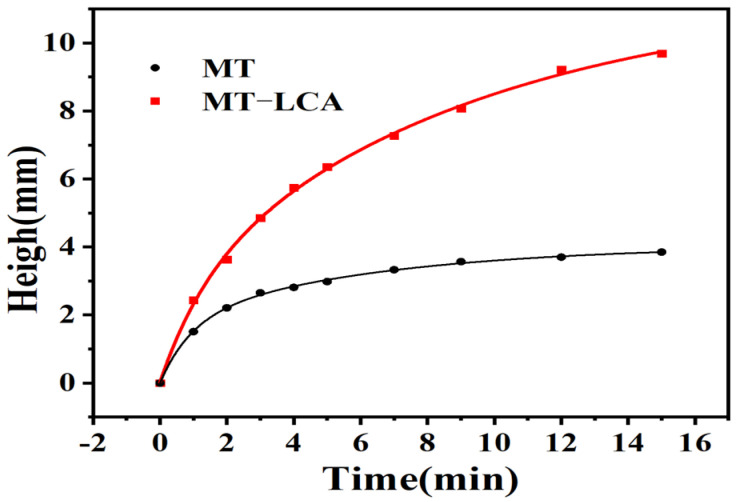
Relationship between height and time of adsorption of soybean oil by MT and MT-LCA.

To explore the directional transport performance of MT and MT-LCA for solvents, the transport distance of soybean oil (Sudan III staining) in MT and MT-LCA was tested at different time intervals. Figure 10 and Figure 11 illustrate the transport capabilities of MT and MT-LCA for soybean oil, and experimental results indicate a faster transport rate of soybean oil in MT-LCA. As shown in Figure 11, when the time reaches 15 min, not only does MT-LCA adsorb soybean oil more rapidly, but it also achieves a higher adsorption capacity. This phenomenon is primarily attributed to two factors. On the one hand, MT is formed by relatively closed cell stacking, without transport channels between cells. After partial dissolution and swelling in an ionic liquid, the original cell walls of MT are eroded, forming transport channels that facilitate the directional transport of the adsorbent. On the other hand, during the preparation of MT-LCA, a large number of macropores, mesopores, and micropores are generated, leading to increased porosity, surface area, and pore size. The higher porosity, nanoscale micropores, and larger pore size in MT-LCA enhance its capillary force, providing efficient directional transport performance [30,39]. Therefore, through partial dissolution and regeneration of MT to transform it into an adsorbent material with a hierarchical pore structure, excellent directional transport functionality is achieved. MT-LCA exhibits potential for selective directional transport.

## 3. Conclusions

A novel lignocellulosic aerogel, MT-LCA, has been successfully prepared from MT through partial dissolution in an ionic liquid, coagulation in water, freezing in liquid nitrogen and freeze-drying. The MT-LC preserves the original honeycomb-like porous structure in the lateral direction and the regularly oriented pore channel structure in the longitudinal direction. Additionally, smaller micropores were also found in cell walls and in the lumen. The retention of anisotropic structure imparts excellent compressive performance to MT-LCA, while the newly formed micropores enhance its porosity and specific surface area. FT-IR results indicate that MT, after dissolution and coagulation, undergoes no chemical reaction. However, there is a change in the crystalline structure of cellulose, transitioning from cellulose I to cellulose II. Both MT and MT-LCA exhibit a quasi-second-order kinetic process in the adsorption of methylene blue, belonging to chemical adsorption. The Langmuir model is more suitable for describing the adsorption of methylene blue. Both adsorbents show monolayer adsorption, and their effective adsorption sites are uniformly distributed. Adsorption experiments with oil and organic solvents indicate that the saturated adsorption capacity is positively correlated with the density of oil and organic solvents. The higher porosity, nanoscale micropores, and larger pore size enhance the capillary force of MT-LCA, providing efficient directional transport performance. The prepared MT-LCA exhibits outstanding compressive performance and efficient directional transport capabilities, making it suitable for applications in areas that demand high compressive performance and selective directional transport.

## 4. Materials and Methods

### 4.1. Materials

The MT was harvest at Guangdong, China. Ethanol, acetone, petroleum ether and paraffin were obtained from Xilong Chemical Co., Ltd. (Guangzhou, China). Methylene blue and Sudan III were provided by Kermel Chemical Reagent Co., Ltd. Tianjin, China. 3-ethyl-1-methyl- imidazolium acetate ([Emim]OAc, 98%) was purchased from Qingdao Ionike New Material Technology Co., Ltd. (Qingdao, China). Methyl silicone oil and engine oil were supplied by Shanghai Aladdin Biochemical Technology Co., Ltd. (Shanghai, China). Soybean oil was purchased from a local market.

### 4.2. Preparation of Lignocellulosic Aerogel

MT specimens, which were characterized by a density of 0.05 g/mm^3^ and a diameter of 20.0 ± 0.1 mm, were prepared for fabrication of lignocellulosic aerogels. The samples were sliced into a thickness of 21 ± 1.5 mm and subjected to water-bath heating at 60 °C for 20 min. Afterward, the samples underwent rapid freezing in liquid nitrogen, followed by freeze-drying at −81 °C for 48 h to eliminate water content. Subsequently, the freeze-dried samples were immersed in an ionic liquid (EmimOAc) and heated in a vacuum drying oven at 90 °C for 4 h. To eliminate the ionic liquid (IL) from the partially dissolving samples, it was coagulated in distilled water for a duration of 7 days. Following this, the lignocellulosic hydrogels underwent another freezing in liquid nitrogen and were subsequently freeze-dried to yield the Medulla tetrapanacis-derived lignocellulosic aerogels (MT-LCA).

### 4.3. Characterizations

Morphological features of both MT and MT-LCA were observed using a scanning electron microscope (SEM) (Hitachi S-4800, Tokyo, Japan). Chemical structures of MT and MT-LCA were analyzed through Fourier-transform infrared spectroscopy (FTIR) (NEXUS-870, Nicolet, MN, USA). Thermal gravimetric analysis was conducted in a temperature range of 35 to 700 °C under a nitrogen atmosphere using a thermal analyzer (TA Q-600, New Castle, PA, USA). Mechanical compression properties were evaluated using a mechanical testing machine (Jindou AG-X plus, Tokyo, Japan). Specific surface area, pore size distribution, and nitrogen adsorption–desorption isotherms were determined with an automatic specific surface area and pore analyzer (Quantachrome AUTOSORB IQ, Boynton Beach, FL, USA). Crystal structures of MT and MT-LCA were examined using X-ray diffraction with an X-ray diffraction analyzer (Puxi D8 DISCOVER, Shanghai, China).

### 4.4. Density and Porosity

The following Equation was set to measure the density (*ρ*) of MT or MT-LCA.

(1) ρ=M/V

where *M* and *V* are, separately, the mass and the volume of MT or MT-LCA. The porosity (*P*) of t MT or MT-LCA was measured according to the following equation.

(2) $$P=(V_{0}-V_{1})/V_{0}\times 100\%$$

where *V*_0_ is the volume of MT or MT-LCA in natural state, and the *V*_1_ is the absolute dense volume of MT or MT-LCA.

### 4.5. Adsorption of Dye

In order to compare the adsorption capacity of MT and MT-LCA for dyes and to investigate the adsorption mechanism, methylene blue was selected as a model dye for dye adsorption experiments [8]. In the experiment assessing methylene blue adsorption, various factors influencing adsorption performance, including pH value, adsorption time, adsorbent dosage, and initial methylene blue solution concentration, were systematically investigated through individual experiments [36,41]. The experimental procedures are detailed as follows:

Firstly, regarding the influence of pH value, a 100 mg/L methylene blue solution was prepared, and its pH was adjusted using hydrochloric acid and sodium hydroxide. The adjusted pH values ranged from 3 to 9. Samples weighing 5 mg were introduced into 50 mL methylene blue solutions at different pH levels, subjected to 24 h of oscillation in a constant-temperature water bath at 30 °C, and the upper clear liquid was extracted for absorbance measurement using a UV-visible spectrophotometer.

Next, to explore the effect of adsorption time, 5 mg samples were added to 100 mg/L methylene blue solutions, and the mixtures were oscillated in a constant-temperature water bath at 30 °C with a shaking speed of 120 rpm/min. At specified intervals, portions of the methylene blue solution were extracted, and their absorbance was measured using a UV-visible spectrophotometer.

For the investigation into the impact of adsorbent dosage, samples weighing 2 mg, 4 mg, 6 mg, 8 mg, and 10 mg were added to 100 mg/L methylene blue solutions, followed by 24 h of oscillation at 30 °C with a shaking speed of 120 rpm/min. Subsequently, portions of the methylene blue solution from the upper clear liquid were collected for absorbance measurement using a UV-visible spectrophotometer.

Lastly, the effect of the initial concentration of the methylene blue solution was studied by preparing solutions with concentrations of 50, 100, 200, 300, 400, and 500 mg/L. Samples weighing 5 mg were placed in these solutions, and after 24 h of oscillation at 30 °C, the absorbance of the upper clear liquid was measured. The objective was to comprehensively understand the interplay of these factors on the adsorption behavior.

### 4.6. Adsorption Mechanism and Kinetics

The quasi-first-order and quasi-second-order kinetic models were used to investigate the dye adsorption kinetics of MT and MT-LCA [4,8]. The following two groups of formulas represented the quasi-first-order dynamic model and the quasi-second-order dynamic model, respectively:

(3) $$\ln(Q_{e}-Q_{t})=\ln Q_{e}-K_{1}t/2.303$$

(4) $$t/Q_{t}=1/K_{2\;\;}Q_{e}^{2}+1/Q_{e}$$

where *K*_1_ is the rate constant of a quasi-first-order dynamic simulation; *K*_2_ is the rate constant of a quasi-second-order dynamics simulation; *Q_e_* is the adsorption capacity of a sample after adsorption equilibrium; *Q_t_* is the adsorption amount of a sample at time of *t*; *t* is the reaction time.

In order to study the adsorption mechanism of methylene blue on samples, we fitted the experimental data with two isothermal models: Langmuir and Freundlich [4,8]. The equations of the two adsorption isotherms are as follows:

(5) $$\log Q_{e}=\log K_{F}-\log C_{e}/n$$

(6) $$C_{e}/Q_{e}=1/Q_{m\;}K_{L}+{C_{e}/Q_{m}}_{\;}$$

where *C_e_* is the dye concentration at adsorption equilibrium; *Q_e_* is the amount of MB dye adsorbed by the sample at adsorption equilibrium; *K_F_* and *K_L_* are, separately, the adsorption constants of Freundlich and Langmuir; *n* is the empirical constant of adsorption capacity and adsorption strength; *Q_m_* is the maximum adsorption capacity of a single molecular layer.

### 4.7. Oil Sorption Capacity

To further assess the adsorption capabilities of MT and MT-LCA, the saturation adsorption capacities for six common oils and organic solvents were determined. Specifically, soybean oil, engine oil, paraffin oil, ethanol, acetone, and petroleum ether were denoted as SO, MA, PA, ET, AC, and PE, respectively [42]. Prior to sorption, MT and MT-LCA were weighed and immersed in these liquids. To eliminate excess liquid, the oil-loaded MT and MT-LCA were removed and allowed to stand for an additional 1 min, ensuring that any oil on the material surface dripped off due to gravity. The oil sorption capacity (*Kc*) was calculated using the following equation [42].

(7) $$K_{C}={(m}_{1}-m_{0})/m_{0}$$

where *m*_0_ is the sample mass before adsorption, and *m*_1_ is the mass of the sample when oil sorption equilibrium is reached.

## Figures and Tables

**Table 1:** Thermal performance data of MT and MT-LCA.

Samples	T_d5%_/°C	T_Max_/°C
MT	64	166/364
MT-LCA	93	135/364

**Table 2:** Density and compressive mechanical property data of MT and MT-LCA.

Samples	Density (g/cm^3^)	Compressive Modulus (MPa)	Compressive Strength (MPa)
MT	0.050 ± 0.006	5.223 ± 0.121	0.411 ± 0.011
MT-LCA	0.084 ± 0.008	2.484 ± 0.081	0.472 ± 0.051

**Table 3:** Porosities and pore-related physical properties of MT and MT-LCA.

Samples	Porosity (%)	Total Pore Volume (cm^3^/g)	Average Pore Size (nm)	Specific Surface Area (m^2^/g)
MT	38	0.001	36.71	0.669
MT-LCA	51	0.041	22.17	36.576

**Table 4:** Parameters related to dynamic simulation and isothermal model of MT and MT-LCA.

Models		Parameters	MT	MT-LCA
Kinetic adsorption fitting curves	Quasi-first-order kinetic models	*Q_e_*	280.68	223.56
		*K*_1_	0.0048	0.0044
		*R* ^2^	0.90	0.96
	Quasi-second-order kinetic models	*Q_e_*	318.35	263.78
		*K*_2_	0.000024	0.000019
		*R* ^2^	0.95	0.99
Adsorption isotherms	Langmuir	*Q_max_*	425.86	408.15
		*K_L_*	0.040	0.038
		*R* ^2^	0.98	0.95
	Freundlich	*K_F_*	67.56	63.89
		*n*	3.15	3.10
		*R* ^2^	0.84	0.90

## Data Availability

The original contributions presented in the study are included in the article, further inquiries can be directed to the corresponding authors.
